# Iron deposition in substantia nigra: abnormal iron metabolism, neuroinflammatory mechanism and clinical relevance

**DOI:** 10.1038/s41598-017-14721-1

**Published:** 2017-11-02

**Authors:** Zhuo Liu, Hui-cong Shen, Teng-hong Lian, Lei Mao, Shou-xian Tang, Li Sun, Xi-yan Huang, Peng Guo, Chen-jie Cao, Shu-yang Yu, Li-jun Zuo, Xiao-Min Wang, Sheng-Di Chen, Piu Chan, Wei Zhang

**Affiliations:** 1 Department of Neurology, Beijing Tiantan Hospital, Capital Medical University, Beijing, 100050 China; 2 Department of Radiology, Beijing Tiantan Hospital, Capital Medical University, Beijing, 100050 China; 3 Department of Radiology, Beijing Chuiyangliu Hospital, Beijing, 100022 China; 4 Department of Geriatrics, Beijing Tiantan Hospital, Capital Medical University, Beijing, 100050 China; 5 Department of Physiology, Capital Medical University, Beijing, 100069 China; 6 Department of Neurology, Ruijin Hospital Affiliated to Shanghai Jiaotong University School of Medicine, Shanghai, 200025 China; 7 Department of Neurology, Beijing Xuanwu Hospital, Capital Medical University, Beijing, 100053 China; 8 China National Clinical Research Center for Neurological Diseases, Beijing, 100050 China; 9 Center of Parkinson’s Disease, Beijing Institute for Brain Disorders, Beijing, 100053 China; 10 Beijing Key Laboratory on Parkinson’s Disease, Beijing, 100053 China; 11 Key Laboratory for Neurodegenerative Disorders of the Ministry of Education, Capital Medical University, Beijing, 100069 China

## Abstract

Parkinson disease (PD) is associated with multiple factors, including iron, which is demonstrated to deposit excessively in PD brains. We detected iron deposition by susceptibility weighted image (SWI) and measured the levels of iron metabolism-related proteins and inflammatory factors in cerebrospinal fluid (CSF) and serum of PD patients and control subjects. Clinical symptoms of PD were evaluated by series of rating scales. Relationships among above factors were analyzed. Results showed that corrected phase (CP) value of substantia nigra (SN) was significantly decreased in PD group compared to control group, hence, SN was the main region with excessive iron deposition. In PD group, ferritin was significantly elevated in CSF and reduced in serum compared to control group, and levels of ferritin in CSF and serum were both significantly and positively correlated with CP value of SN, thus, abnormal iron metabolism in central and peripheral systems was associated with iron deposition. CP value of SN in PD group was significantly and negatively correlated with interleukin-1β level in CSF, so interleukin-1β might be a neuroinflammatory factor produced by excessive iron in SN. Iron deposition in SN was significantly correlated with motor symptoms and part of non-motor symptoms of PD.

## Introduction

Parkinson disease (PD) is a common neurodegenerative disorder, which is mainly characterized by motor symptoms, and is often accompanied by an array of non-motor symptoms, such as psychiatric symptoms, sleep disorders, autonomic dysfunctions and abnormal sensation, etc^1^. Etiologically, PD has multiple risk factors, including aging, genetic predisposition and environmental toxins^2^. Iron is demonstrated to deposit excessively in PD brains and can be detected by susceptibility weighted imaging (SWI). Iron accumulation is a metabolic lesion that may be a result of genetics, fatigue of iron transport machinery with aging, and inflammation in brain etc, or iron may accumulate in the pathogenic mechanism of PD^3–6^. However, the underlying mechanism of over deposition of iron in PD brain remains unknown.

Currently, the pathogenesis of PD remains uncertain. Mounting evidences reveal that neuroinflammation featured by microglial activation plays a pivotal role on the development of PD and drives the progressive degeneration of dopaminergic neurons as an engine^7^. In our previous *in vitro* study^8^, activated microglia by iron released a large amount of neuroinflammatory factors, such as superoxide, intracellular reactive oxygen species (iROS), hydrogen peroxide (H_2_O_2_), nitric oxide (NO), interleukin (IL)-1β, tumor necrosis factors (TNF)-α and prostaglandin (PG) E_2_, potentiating progressive dopaminergic neurodegeneration. Nevertheless, in PD patients, the relationships between iron deposition in SN, the clinical motor symptoms and non-motor symptoms haven’t been well elucidated. Moreover, it remains unknown what a role of neuroinflammatory mechanism plays on the relationship between iron deposition in SN and clinical symptoms.

In PD patients and control subjects recruited in this investigation, iron deposition was detected by susceptibility weighted image (SWI), the levels of iron metabolism-related proteins and inflammatory factors in cerebrospinal fluid (CSF) and serum were measured, motor symptoms and non-motor symptoms of PD patients were evaluated by a body of rating scales, and the relationships among the factors above were analyzed eventually.

## Results

**Figure 1:**
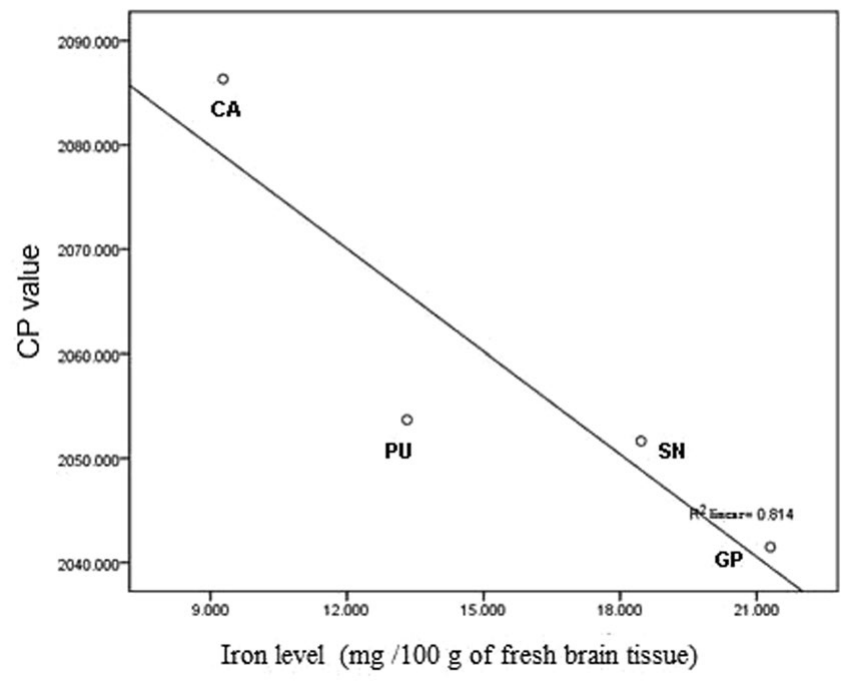
Correlation between the CP values and iron levels in SN, caudate nucleus, putamen and globus pallidus in normal elderly (r = −0.902, P = 0.043) . Pearson correlation was used for the analyses. CP = corrected phase, SN = substantia nigra.

In control group, the CP values of SN, caudate nucleus, putamen and globus pallidus detected by SWI were 2051.64, 2086.32, 2053.69 and 2041.49, respectively, which were all significantly and negatively correlated with iron level according to the technique set up by Hallgren^9^ (r = −0.902, P < 0.05) (Fig. 1).

The CP values of SN, caudate nucleus, putamen and globus pallidus in PD and control groups are shown in Table 1. The CP value of SN in PD group was significantly decreased compared to control group (P < 0.01), whereas the CP values of caudate nucleus, putamen and globus pallidus were not significantly different.

**Table 1:** The CP values in SN, caudate nucleus, putamen and globus pallidus in PD group and control group (mean ± SD).

	Control group n = 30	PD group n = 60	P
SN	2051.64 ± 31.56	2029.63 ± 21.06	0.000**
caudate nucleus	2086.31 ± 15.98	2081.59 ± 8.36	0.525
putamen	2053.69 ± 37.38	2055.64 ± 15.50	0.639
globus pallidus	2041.49 ± 39.77	2051.02 ± 22.22	0.112

Two-tailed t test was used for the comparison. CP = corrected phase, SN = substantia nigra. **P < 0.01.

The levels of iron metabolism-related proteins in CSF in PD and control groups are presented in Table 2. Ferritin level in CSF in PD group was significantly elevated compared to control group (P < 0.05). However, there was no significant difference between the levels of transferrin and lactoferrin in CSF between PD and control groups. Further correlation analyses demonstrated that the CP value of SN was significantly and positively correlated with ferritin level in CSF (r = 0.619, P < 0.05). However, the CP value of SN was not correlated with the levels of transferrin and lactoferrin in CSF in PD group. Pearson correlation analyses were performed between ferritin levels in serum and CSF. Ferritin level in serum was significantly and positively correlated with that in CSF (r = 0.551, P < 0.05).

**Table 2:** The levels of iron metabolism-related proteins in CSF in PD group and control group (mean ± SD).

	Control group n = 20	PD group n = 13	P
ferritin (ng/ml)	2.75 ± 0.65	3.71 ± 1.54	0.025*
transferrin (μg/ml)	0.17 ± 0.26	0.15 ± 0.03	0.122
lactoferrin (μg/ml)	126.79 ± 95.50	131.50 ± 74.78	0.883

Two -tailed t test was used for the comparison. *P < 0.05.

The levels of iron metabolism-related proteins in serum in PD and control groups were shown in Table 3. Ferritin level in serum in PD group was evidently decreased compared to that in control group (P < 0.05). There was no significant difference in the levels of transferrin and lactoferrin in serum between PD and control groups. Further correlation analyses demonstrated that the CP value of SN was significantly and positively correlated with ferritin level in serum. However, the CP value of SN was not correlated with the levels of transferrin and lactoferrin in serum in PD group.

**Table 3:** The levels of iron metabolism-related proteins in serum in PD group and control group (mean ± SD). Two-tailed t test was used for the comparison. *P < 0.05.

	Control group n = 30	PD group n = 35	P
ferritin (ng/ml)	9.80 ± 4.08	7.77 ± 2.84	0.022*
transferrin (μg/ml)	4.47 ± 0.75	4.26 ± 1.14	0.392
lactoferrin (μg/ml)	62.37 ± 29.33	71.93 ± 33.15	0.384

The correlations between iron deposition in SN and an array of neuroinflammatory factors in CSF of PD group were analyzed in Table 4. The CP value of SN had a significantly negative correlation with the level of IL-1β in CSF (r = −0.587, P < 0.05). The CP value of SN was not correlated with the levels of H_2_O_2_, NO, TNF-αand PGE_2_ in CSF.

**Table 4:** Correlations of the CP value in SN with the levels of neuroinflammatory factors in CSF in PD group. Pearson correlation was used for the analyses. *P < 0.05.

	r	P
IL-1β (pg/ml)	−0.587	0.035*
H_2_O_2_ (pg/ml)	0.069	0.822
NO (pg/ml)	0.075	0.808
PGE_2_ (pg/ml)	−0.476	0.100

The relationships between iron deposition in SN and motor symptoms of PD were investigated. The result indicated that the CP value in SN was significantly and negatively correlated with the score of UPDRS III in PD group (r = −0.615, P < 0.05).

The relationships between iron deposition in SN and a variety of non-motor symptoms of PD were presented in Table 5. The CP value in SN was significantly and negatively correlated with the number of non-motor symptoms. Further analysis indicated that the CP value in SN was significantly and positively correlated with MoCA score. The CP value in SN was significantly and negatively correlated with the scores of PSQI and SCOPA-AUT in PD group.

**Table 5:** Correlations of the CP value in SN and the numbers and scores of non-motor symptoms in PD group. Pearson correlation was used for the analyses. MoCA = Montreal Cognitive Assessment; MMSE = Mini-Mental State Examination; PSQI = Pittsburgh Sleep Quality Index; ESS = Epworth Sleepiness Scale; SCOPA-AUT = Parkinson’s Disease-Autonomic Questionnaire; HAMD = Hamilton Depression; HAMA = Hamilton Anxiety ; FS-14 = Fatigue Scale-14; RLSRS = Restless Legs Syndrome Severity Rating Scale. *P < 0.05.

	r	P
number of non-motor symptoms	−0.732	0.020*
MoCA score	0.789	0.049*
MMSE score	−0.199	0.207
PQSI score	−0.634	0.046*
ESS score	0.166	0.293
SCOPA-AUT score	−0.642	0.012*
HAMD (24 items)score	−0.046	0.772
HAMA (14 items)score	0.016	0.921
FS-14 score	0.149	0.346
RLSRS score	0.830	0.601

## Discussion

Iron deposition in brain can be reflected by the CP value detected by SWI. The CP value is negatively correlated with iron level in brain, which means that the lower the CP value, the higher the iron level^10–12^. In this study, the CP values of SN, caudate nucleus, putamen and globus pallidus detected by SWI in normal elderly were significantly and negatively correlated with the iron level in the brain regions above reported by autopsy, consistent with the results from several previous studies^13–15^. Thus, SWI can be a reliable and noninvasive technique for detecting iron level in brain.

Previous studies on the histopathology and autopsy suggested that iron deposited excessively in SN, which was revealed by an increase of 25–100% in total iron level^16^ and elevation of 225% in ferrous ion^17^. However, other investigations on PD patients displayed that iron levels in caudate nucleus and globus pallidus were increased compared to that in control group. A recent study showed a markedly elevated iron level in caudate nucleus, which was helpful for the diagnosis and longitudinal monitoring of PD^18^. In this study, PD patients presented significantly decreased CP value in SN, whereas caudate nucleus, putamen and globus pallidus displayed no significantly change of CP value compared to control group. These results demonstrate that SN is the main brain region with excessive iron deposition in PD patients.

Even in the early stage of PD, iron in brain begins to redistribute with its metabolism disturbed^19^, while the total iron remains unchanged. It was found that iron level in the neurite around neuron was remarkably elevated in PD brains^20^. In this study, ferritin level in CSF in PD group was obviously increased, suggesting that the disturbed iron metabolism mainly involved ferritin. Ferritin is divided into heavy-ferritin (H-ferritin) and light-ferritin (L-ferritin) based on its structure. H-ferritin converts higher toxic Fe^2+^ to lower toxic Fe^3+^ and participates in the absorption and utilization of iron, whereas L-ferritin contributes to the long-term storage of iron.

There are both H-ferritin and L-ferritin in glial cells, and ferritin is the main storage form of iron in astrocytes. Ferritin in astrocytes not only reduces the toxic effect of excessive iron, but also serves as the source of iron supplement when iron level is reduced^8,21^. Therefore, we speculate that the increased ferritin level in CSF might be mainly from astrocytes.

Iron in gastrointestinal tract is absorbed into blood, but it does not enter brain through blood-brain barrier. Iron combines with transferrin and then enters cells via transferrin receptor-mediated endocytosis. Iron is released by divalent metal transporter-1 (DMT-1) and then either enters mitochondria or converts to ferritin for storage^22^. In this study, ferritin level in serum of PD group was drastically decreased compared to control group; however, ferritin level in serum was far higher than that in CSF in PD group. These results supported an iron trafficking lesion that might lead to the loss of iron in blood and consequent accumulation of iron in brain.

In the normal condition, the total amount of ferritin distributed in both CSF and parenchyma of brain is fixed. However, the ratio of the ferritin in CSF and parenchyma may change with PD progression. Data from other investigator showed that ferritin level in brain was already increased in the early stage of PD, and the binding activity of ferritin with iron, especially in SN, increased the risk of PD progression^23^, which gradually decreased ferritin level in CSF. In this study, the CP value of SN in PD group had a significantly positive correlation with ferritin level in CSF, indicating that ferritin level in CSF was remarkably decreased with more iron deposition in SN. Meanwhile, the CP value of SN in PD group was significantly and positively correlated with ferritin level in serum, suggesting that the more the iron depositions in SN, the lower the ferritin level in serum. The detailed mechanism underlying the relationship between ferritin level in serum and iron deposition in SN needs to be furtherly explored in the future.

In this study, UPDRS III score was significantly and negatively correlated with the CP value in SN of PD patient, indicating that the severer the motor symptoms, the more the iron depositions in SN. Meanwhile, the CP value of SN was significantly and negatively correlated with IL-1β level in CSF, suggesting that the excessively accumulated iron in SN of PD patients might cause neuroinflammation indicated by robust production of IL-1β^24^. Neuroinflammation characterized by microglial activation and the generation of a line of neuroinflammatory factors is crucial to dopaminergic neurodegeneration in PD^25^. Microglia in SN are five times more than that in other brain regions, thus they are highly sensitive to a variety of factors and readily activated upon stimulations. It was observed that ferritin level in SN was significantly increased in PD patients^8^ and more ferrous ion were released from ferritin. Our previous study illustrated that ferrous ion induced progressive dopaminergic neurodegeneration through dual mechanisms of causing direct neurotoxicity and enhancing the neurotoxicity via neuroinflammation^8^. These might result in the depletion of dopamine in striatum, and eventually, the deterioration of motor symptoms. Thus, iron deposition in SN might be closely correlated with motor symptoms through neuroinflammation in PD patients. A recent research pointed out deferiprone significantly reduced labile iron and biological damage in oxidation-stressed cells and animals, improving motor functions while raising striatal dopamine^26^.

Currently, there are very few studies reporting the relationships between iron deposition and non-motor symptoms of PD^27^. In this study, iron deposition in SN was related to several non-motor symptoms of PD. First of all, the CP value in SN  was significantly and positively correlated with MoCA score, a sensitive rating scale for mild cognitive impairment (MCI) of PD, suggesting that PD with MCI might be relevant to iron deposition in SN. It could be very useful to extend the exploration also to other subcortical structures, such as hippocampus, etc, which are more relevant to cognitive function. Furthermore, the iron deposition in SN was significantly associated with sleep disorders and autonomic dysfunction, which mechanism hasn’t been well investigated. Dopaminergic neurons are regulated by many other neuronal systems. Complex interactions between dopamine and other neurotransmitter systems can affect a variety of human functions. For example, rapid eye movement sleep behavior disorder, a common sleep disorder of PD, was related to the decreased activity of dopamine receptor and increased activity of acetylcholine receptor^7^. Thus, the depletion of dopamine and the imbalance between dopamine and other neurotransmitters might be relevant to the non-motor symptoms of PD^28^.

In this investigation, the correlation between the CP value in SN and clinical symptoms is very interesting. Other studies found the correlation between the level of ferritin and symptoms of PD. In a Parkinson’s clinical trial with deferiprone, an iron chelator, ferritin was observed to be increased in PD subjects, and deferiprone improved clinical symptoms of PD, which coincided with the restoration of ferritin level^26^.

In summary, SN is the main brain region with excessive iron depositions in PD patients. Abnormal iron metabolism in the central and peripheral systems indicated by the increase of ferritin level in CSF and decrease of ferritin level in serum is associated with iron deposition in SN of PD patients. IL-1β may be a neuroinflammatory factor produced by the accumulated iron in SN of PD patients. Iron deposition in SN is related to both motor symptoms and several non-motor symptoms of PD, including cognitive impairment, sleep disorders and autonomic dysfunction. Thus, inhibition of neuroinflammation induced by the excessively deposited iron in SN may be a novel therapeutic target for PD.

Below are the limitations of the study. Firstly, SWI technique is not exclusively sensible to iron, which also identifies deoxyhaemoglobin, ferritin, hemosiderin and calcium^29^. Secondly, CSF samples were relatively insufficient, since it was really difficult to obtain CSF samples from those PD patients who were old, combined with low intracranial pressure, spinal deformity and bone hyperplasia, etc. Thirdly, in order to confirm and further expand these findings, replication in bigger cohorts of PD patients and detection of iron deposition in other structures involved in cognition, such as hippocampus, are needed in the future.

## Methods

### Standard protocol approvals, registrations, and patient consents

This study was approved by Beijing Tiantan Hospital Review Board. Written informed consents were obtained from all subjects in this study. All experiments were performed in accordance with relevant guidelines and regulations.

## Subjects

### Patients with PD

United Kingdom Parkinson Disease Society Brain Bank criteria^30^ was used for PD diagnosis. Total 60 PD patients were consecutively recruited from the Department of Neurology and Geriatrics, Beijing Tiantan Hospital, Capital Medical University. Demographic variables of participants were listed in Table 6.

**Table 6:** Demographic variables of control group and PD group. Two-tailed t test was used for the comparison for age and x^2^ test was used for the comparison for sex. NA = not available.

	Control group n = 30	PD group n = 60	P
age (years, mean ± SD)	57.27 ± 9.31	62.34 ± 9.60	0.092
male/total [cases/total (%)]	16/30 (53.3%)	34/60 (56.7%)	0.553
disease duration [years, mean ± SD]	NA	4.62 ± 5.18	
clinical phenotype [cases/total (%)]	NA		
tremor type	NA	6/60 (10.0%)	
rigidity-bradykinesia type	NA	5/60 (8.3%)	
mixed type	NA	49/60 (81.7%)	

### Control participants

Total 30 age and sex-matched control subjects were all healthy people. They were recruited based on the following criteria: (1) no neurological symptoms and positive signs; (2) no essential tremor, PD, secondary parkinsonism or Parkinson plus syndrome; (3) no encephalitis, meningitis, cerebrovascular diseases, brain tumors and other intracranial diseases; (4) no inflammatory, infectious and autoimmune diseases in peripheral and central systems; (5) no blood donation history; (6) female controls should had been through menopause; (7) no brain trauma and surgical histories; (8) no dysarthria or mental illness which affect expression.

### Clinical assessments of PD patients

Severity of PD was assessed by Hoehn-Yahr (H-Y) Stage. Motor symptoms of PD were evaluated by Unified Parkinson’s Disease Rating Scale (UPDRS) III.

Non-motor symptoms were assessed by series of rating scales, including Non-motor Symptoms Questionnaire (NMSQ) for overall evaluation, Montreal Cognitive Assessment (MoCA) and Mini-Mental Status Examination (MMSE) for cognitive impairment, Pittsburgh Sleep Quality Index Scale (PSQI) for decline of sleep quality, Epworth Sleepiness (ESS) for daytime sleepiness, Scale For Outcomes in PD For Autonomic Symptoms (SCOPA-AUT) for autonomic dysfunction, Hamilton Anxiety (HAMA)-14 items for anxiety, Hamilton Depression Scale (HAMD)-24 items for depression, Fatigue Scale (FS)-14 and Fatigue Severity Scale (FSS) for fatigue and its severity, and Restless Legs Syndrome Severity Rating Scale (RLSRS) for restless legs symptoms.

### Detection of iron deposition in brain by SWI

Participants were scanned by a 3.0 T scanner (Magnetom Trio Tim, Siemens Medical Systems, Germany) equipped with an eight-channel head coils. SWI image was obtained parallel to the anteroposterior commissural line by using a sequence with the following parameters: TR/TE = 20/28ms; FA = 15°; slice thickness = 1.2 mm; field-of-view (FOV) = 320 × 240, matrix = 320 × 221^10^.

SWI images were reviewed by experienced radiologists in order to rule out brain lesions. SWI phase images, magnetic moment images, SWI images and minimum intensity projection (MIP) images were all reviewed online on Leonardo workstation. SWI source data were post-processed by using SPIN (signal processing in NMR) software (Detroit, Michigan) to detect and quantitate the corrected phase (CP) value in the region of interest (ROI), including SN, caudate nucleus, putamen and globus pallidus. The size of ROI depended on the size of the deep brain gray matter nuclei. CP value was calculated by the professionally trained and experienced doctors who were blind to grouping. Calcification and visible lesions were avoided during the measurement.

### Collections of CSF and serum samples

Before fasting, CSF (3 mL in a polypropylene tube via lumbar puncture) and serum (2 mL venous whole blood) were collected from PD patients whose conditions allowed us to withdraw the antiparkinsonian drugs for 12 to 14 hours. CSF samples of 13 patients and 20 controls and serum samples of 35 patients and 30 controls were collected. We aliquoted a 0.5 mL volume of CSF and serum into separate cryotubes (Nunc), which we kept frozen at −80 °C until ready for assay, with separate aliquots for each measure to avoid repetitive freeze-thawing and potential degradation of protein. Clinical assessments and sample collections from PD patients were all performed during off phase.

### Measurements of iron metabolism-related proteins in CSF and serum

The levels of iron metabolism**-**related proteins, including ferritin, transferrin and lactoferrin in CSF and serum from participants were detected by Enzyme Linked Immunosorbent Assay (ELISA). Ab108837 kit for ferritin and ab108911 kit for transferrin were from Abcam (British). E01L0224 kit for lactoferrin was from Shanghai LanJi Biotechnology Limited Company (China).

### Measurements of neuroinflammatory factors in CSF and serum

The levels of H_2_O_2_ and NO in CSF and serum from participants were detected by chemical colorimetric method. A064 kit for H_2_O_2_ and A012 kit for NO were all from Nanjing Jiancheng Biological Engineering Research Institute (China). The levels of TNF-α, IL-1β and PGE_2_ in CSF and serum from participants were detected by ELISA. All kits were from Bender Company (Germany).

### Data analyses

SPSS Statistics 20.0.0 (IBM Corporation, Armonk, NY) was used for statistical analyses. Analyses included the comparisons of the levels of iron metabolism-related proteins and neuroinflammatory factors in CSF and serum between PD and control groups.

We presented continuous variables, if normally distributed, as means ± SDs and compared the 2 groups using a 2-tailed t test. If not normally distributed, we presented continuous variables as median (quartile) and compared them by using a nonparametric test. Discrete variable comparisons were performed by using x^2^ test.

Pearson correlation analyses were conducted among CP value in SN, the levels of iron metabolism**-**related proteins and neuroinflammatory factors in CSF and serum, and the scores of motor symptoms and non-motor symptoms in PD group.
